# A Giant Pseudoaneurysm of the Forearm as Unusual Complication of Bacterial Endocarditis

**DOI:** 10.1155/2013/549529

**Published:** 2013-09-23

**Authors:** Michele Arcopinto, Teresa Russo, Antonio Ruvolo, Antonio Cittadini, Luigi Saccà, Raffaele Napoli

**Affiliations:** Department of Translational Medical Sciences, School of Medicine, Federico II University, 5 Via Sergio Pansini, 80131 Napoli, Italy

## Abstract

A 59-year-old man with fever was diagnosed with endocarditis due to *Streptococcus bovis*. Two weeks after antibiotic therapy was started, he presented with red and painful swelling of the forearm without any sign of systemic inflammation. A giant hematoma connected to the radial artery was detected with ultrasound. Surgical intervention with the removal of multiple, sterile clots from the hematoma was performed, and the multiple lacerations of the artery detected were corrected. This is the first case reporting rupture of the radial artery as a complication of infective endocarditis.

## 1. Background

Endocarditis is a potentially life-threatening inflammation of the inner layer of the heart, mainly involving epithelium of the mitral valve. In addition to local damage and secondary hemodynamic impairment, endocarditis could cause a wide array of systemic and/or organ-specific complications with several mechanisms [1]. In the current paper, we describe the sudden development of a giant pseudoaneurysm of the forearm due to rupture of the radial artery in a patient with bacterial endocarditis diagnosed two weeks earlier and the surgical repair of the artery wall.

## 2. Case Description

**Figure 1:**
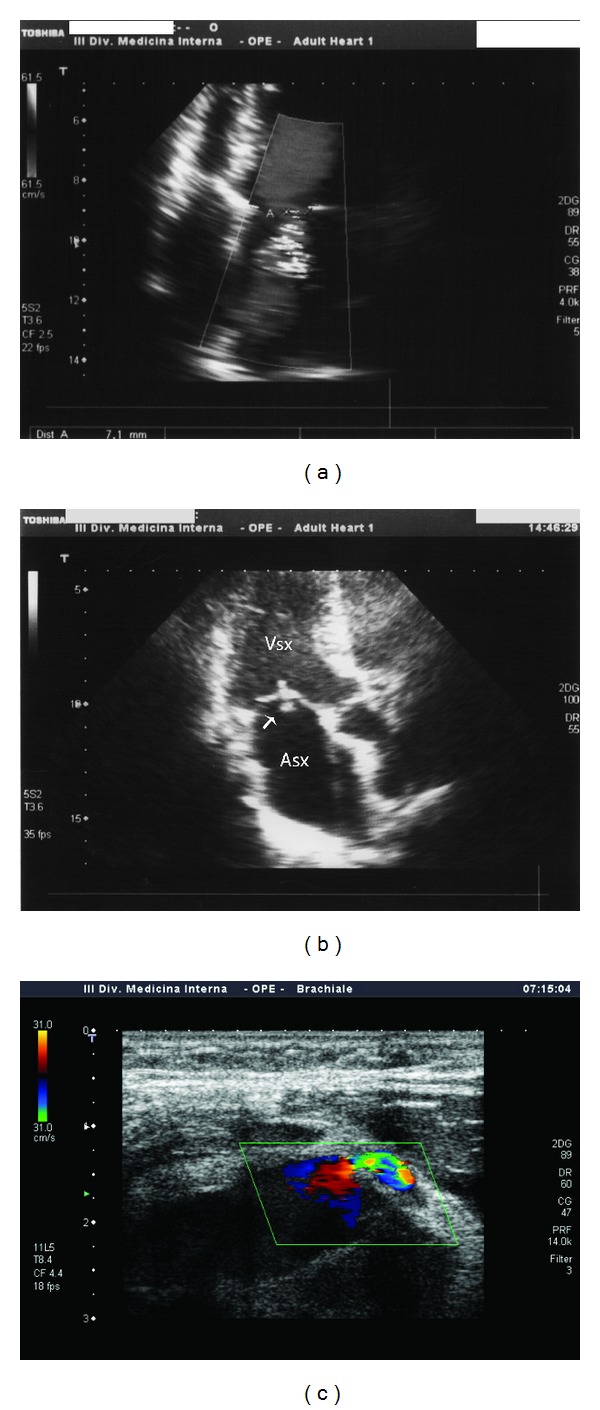
(a) Color Doppler imaging showing mild-to-moderate mitral regurgitation; (b) three-chamber apical view showing vegetation on distal third of mitral anterior leaflet; (c) echography of anterior aspect of right forearm showing color Doppler imaging of radial artery refueling perivascular blood collection.

**Figure 2:**
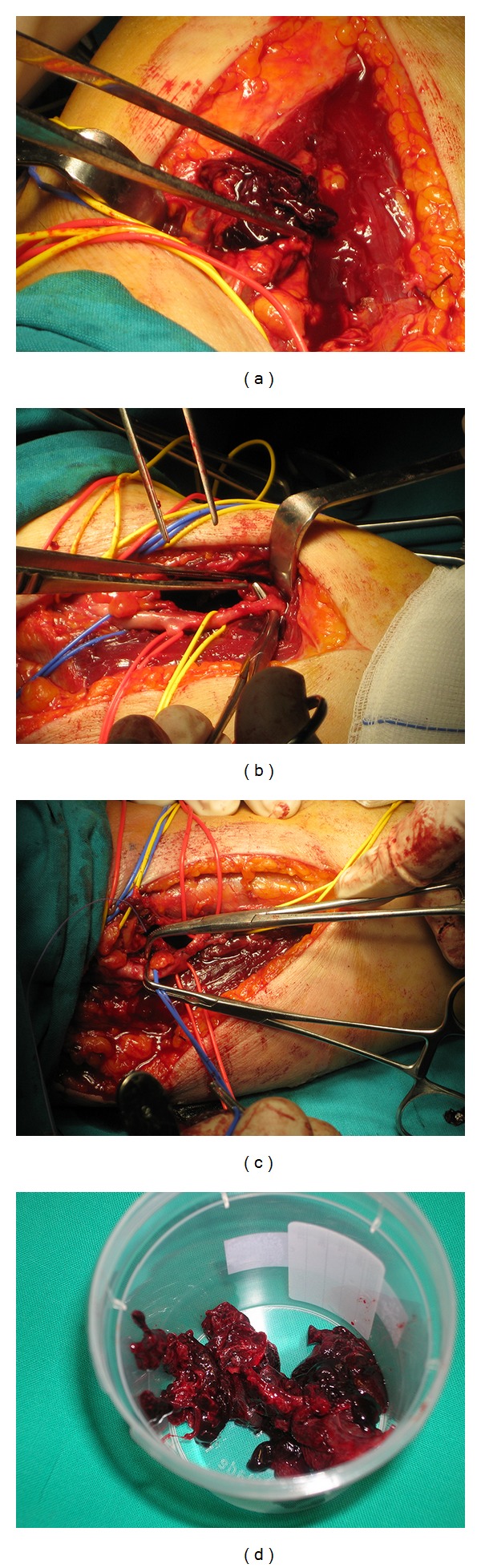
Consecutive phases of vascular intervention. (a) Clot detection immediately below muscular fascia; (b) isolation of vessels and recognition of multiple and point lacerations on the anterior and medial sides of radial artery; (c) radial arterial repair with application of 6-0 prolene sutures; (d) thrombus removed from periarterial collection.

A 59-year-old man with no history of major diseases was admitted to the hospital for the presence of fever during the previous 8 weeks, asthenia, myoarthralgia, and abdominal pain. Before being hospitalized, the patient was prescribed an offhand antibiotic therapy (amoxicillin 2 g/die for five days) without any improvement of his symptoms. On arrival, the physical examination performed showed no clear sign of disease, except for a mild systolic murmur detectable at the cardiac apex. Body temperature was between 37.5 and 38.7°C during the day, heart rate was 90 bpm, respiration rate 20/min, and blood pressure 130/80 mmHg. Blood check showed mild neutrophilic leukocytosis, high C-reactive protein and fibrinogen. Chest radiogram and urinalysis were normal. During the following days, three consecutive venous blood samples were taken one day apart from each other for culture. In these blood cultures *Streptococcus bovis,* was constantly present. Transthoracic echocardiography showed vegetations on the anterior leaflet of the distal third of the mitral valve, associated with moderate regurgitation. According to modified Duke criteria [2], diagnosis of bacterial endocarditis was established and treatment with ceftriaxone (2 g IV, daily), based on actual bacterial susceptibility, was started and continued for the following 4 weeks. Right from the first week of treatment, the blood cultures were negative, fever disappeared, and the clinical conditions improved. The patient was then discharged. Two weeks after diagnosis, the patient returned to the hospital with a giant, red, and painful swelling on the anterior side of the left forearm (about 10 × 12 cm). This swelling appeared suddenly and was associated with rise in body temperature for a few days. No signs of ischemia in the left hand were present. Laboratory data showed an increase of inflammatory markers (C-reactive protein and erythrocyte sedimentation rate), whereas two consecutive blood cultures were negative for germs. A major trauma as a possible explanation of the forearm swelling was easily ruled out with careful patient's interview. An echography of the forearm was performed and an ~8 cm, low-echogenic, blood collection connected with the radial artery was clearly visible (Figure 1). On the basis of the results of the antibiotic testing on the *Streptococcus bovis* isolated in the original blood culture, medical therapy was upgraded with Gentamicin, 1 mg/kg every 8 hours. To stop the refueling of the pseudoaneurysm by the artery, since the surgical support was not immediately available, two consecutive 20-minute compressions of the forearm were applied, but the results were unsatisfactory. Therefore, the patient underwent surgery for diagnostic and therapeutic purposes. During the surgery, after the incision of the brachial fascia, a big clot was evident. Several lacerations on different sides of radial artery were also visible, providing possible explanation for the blood extravasation. No vegetations or other major abnormalities of the arterial wall were found. The vessel integrity was then reconstructed with excision of the damaged segments and primary end-to-end anastomosis of the mobilized extremities with application of 6-0 prolene sutures was performed (Figure 2). Microbiological examination of the tissues removed showed complete sterility. After two additional weeks of antibiotic treatment, medical therapy was discontinued and a strict follow-up was planned. Thereafter, the patient has been checked several times till a year later and no recurrence of systemic or local signs or symptoms has been detected. Radial arterial patency and hand hemodynamics have been assessed through serial ultrasound examinations of ulnar and radial arteries. As *S. bovis* is known to be associated to colonic neoformations, we carried out also a screening colonoscopy, and four adenomatosis masses were removed from the gut.

## 3. Discussion

Complications of endocarditis are quite frequent and severe despite the progress made in antibiotic therapy and cardiac surgery [3]. Vascular complications are generally due to septic embolization from valve vegetations with subsequent thrombosis or hemorrhage in target organs. The most often observed complication in left-sided native valve endocarditis is cerebral ischemia due to septic embolization. Hemorrhagic strokes are less frequent and account for 12–30% of neurologic complications [4]. Intracranial hemorrhage in the setting of infective endocarditis recognizes different pathophysiologic mechanisms: transformation of primary ischemic lesions, formation and rupture of mycotic aneurysms, and rupture of intraparenchymal vessels due to necrotizing arteritis [5]. Although vascular complication of infective endocarditis might involve virtually every district in the body, far fewer cases of extracerebral arterial complications have been reported. In a multicenter prospective study including 384 cases of definite infective endocarditis, 7.3% of patients developed embolic complications after initiation of antibiotic therapy, with total embolic events (before and after antibiotic therapy) occurring in 34.1% of them [3]. Among 28 cases of complication during therapy, common sites of embolization were central nervous system (14 cases), spleen (9 cases), and peripheral artery (5 cases). Excluding vascular disease presenting as the first symptom of subclinical endocarditis, 71.4% of complications occurred in the first 15 days of adequate antibiotic treatment (median time of 7 days).

We describe the case of a patient with the rupture of radial artery secondary to subacute bacterial endocarditis sustained by *S. bovis*. As the clinical presentation of vascular involvement was atypical (no clinical findings of ischemia downstream the vascular bed), possible differential etiology including preexisting vasculitis or autoimmune diseases was considered, but no history or signs were found.

Local predisposing factors, such as previous trauma, prior surgical interventions, or professional, chronic, and mechanical solicitation, were also ruled out. Timing of onset was well in agreement with previous reports of vascular complications occurring under antibiotic therapy (14 days), but some novel aspects were present in this case. First of all, the rupture of the radial artery has not yet been reported as complication of infective endocarditis. Secondly, sterility of the clot removed during the surgical procedure makes the interpretation of arterial injury further challenging. Classic mechanisms postulated for cerebral hemorrhagic lesions, mainly mediated by aneurysm formation and rupture, do not apply in our case. Although, the patient had a low cardiovascular risk profile, and radial artery is a district poorly affected by atherosclerotic alterations, we cannot rule out preexisting, subclinical plaques of the artery tract before overlying events. A prior lesion may have served as predisposing condition for metastatic bacterial colonization and then for parietal infection/inflammation with subsequent vulnerability to physiologic mechanical stress. As we found sterile field at the moment of surgical artery repair, inflammation might self-perpetuate even in the absence of antigenic stimulation. Two conditions make this multistep hypothesis reliable: (1) *S. Bovis* is considered among germs with the highest risk for embolization [3, 6]; (2) time between endocarditis diagnosis and arterial rupture is consistent with subclinical embolization in the first days after antibiotic therapy initiation (period at the highest risk for embolism [7]) and may be considered sufficient for the subsequent damage of the arterial wall induced by inflammation. However, since the patient was on proper, effective, antibiotic therapy and the vascular tissues removed by the surgeon were sterile, damage to the vascular wall must have been triggered before the therapy become effective to eliminate bacteria. To our knowledge, this report describes, for the first time, a case of endocarditis complicated by the rupture of radial artery. Vascular damage, although probably initiated by microbial infection, can become insensitive even to effective antibiotic therapy and lead to dangerous complications, particularly when involving vital organs.

## References

[1] Habib G, Hoen B, Tornos P (2009). Guidelines on the prevention, diagnosis, and treatment of infective endocarditis (new version 2009): the task force on the prevention, diagnosis, and treatment of infective endocarditis of the European society of cardiology (ESC). *European Heart Journal*.

[2] Li JS, Sexton DJ, Mick N (2000). Proposed modifications to the Duke criteria for the diagnosis of infective endocarditis. *Clinical Infectious Diseases*.

[3] Thuny F, Disalvo G, Belliard O (2005). Risk of embolism and death in infective endocarditis: prognostic value of echocardiography—a prospective multicenter study. *Circulation*.

[4] Corral I, Martín-Dávila P, Fortún J (2007). Trends in neurological complications of endocarditis. *Journal of Neurology*.

[5] Masuda J, Yutani C, Waki R, Ogata J, Kuriyama Y, Yamaguchi T (1992). Histopathological analysis of the mechanisms of intracranial hemorrhage complicating infective endocarditis. *Stroke*.

[6] Pergola V, Di Salvo G, Habib G (2001). Comparison of clinical and echocardiographic characteristics of *Streptococcus bovis* endocarditis with that caused by other pathogens. *The American Journal of Cardiology*.

[7] Vilacosta I, Graupner C, San Román J (2002). Risk of embolization after institution of antibiotic therapy for infective endocarditis. *Journal of the American College of Cardiology*.

